# Chicago Medical Society—Stated Meeting, July 2

**Published:** 1888-10

**Authors:** 


### CHICACO MEDICAL SOCIETY.

STATED MEETING—July 2, 1888.

The President, J. H. ETHERIDGE, M.D., in the chair.

Dr. J. G. KIERNAN read a paper entitled, "The Mental Symptoms of Heart Disease as an Element in Prognosis, as Illustrated in the Case of Matthew Arnold."

Very frequently an account of death from cardiac failure is preceded by the statement that the deceased was in "unusually high spirits just previous to death, which occurred after unusual exertion." Cases of this kind occurring so frequently naturally raise the question whether this "high spirits" be not the expression of a pathological state. To answer the question is not easy.

Griesinger many years ago called attention to the fact that an emotional variability existed in many sane persons with cardiac disease. Very little attention has been paid to this observation, which would be somewhat astonishing, since the state described is far from infrequent, were it not for the fact that mental phenomena arising from ordinary somatic disease are very little studied—in fact, are pretty generally ignored. To form any idea of the influence of cardiac disease we are therefore forced to study the insane, since the mental phenomena arising in them from any given somatic cause are simply exaggerations of minor mental phenomena of the same type found in the sane affected with the same disease.

For example, Griesinger speaks of a slight emotional mobility which is peculiar to many healthy persons affected with heart disease.

The relations between insanity and cardiac disease are, like the other somatic relations of insanity, three-fold in character: The cardiac disease produces insanity; it is affected by insanity, and it affects pre-existing insanity. The first question naturally arising for consideration is the influence of cardiac disease in the production of insanity. This was much over-estimated by Nasse and the early alienists In a general way it may be said that the types of insanity resulting from cardiac diseases are usually characterized by suspicion and emotional mobility.

Griesinger says that a certain etiological influence must always be attributed to cardiac disease, but there is no special frequency in the occurrence of this influence which shows itself in an exaggeration of the normal emotional mobility of cardiac disease in suspicion and depression. Luys says that the symptoms of the psychoses arising from cardiac affections are excitement and loquacity, followed by more or less prolonged somnolence. In certain cases when the cardiac affection shows itself in periodic attacks of dispnœa with extreme respiratory anxiety, there is an equal periodicity in the appearance of psychical symptoms which show themselves at the height of the respiratory anxiety and disappear with it. Hertz has had similar experience. Burman, Savage, and Dickson have noted similar phenomena, and these are also laid stress upon by Mickle in his recent lecture.

P. Macdowell says, "We may accept it as a general, though not a constant, rule, that mitral disease produces symptoms of melancholia, and aortic lesions more frequently those of a maniacal type."

Dr. Alice Bennett has found that emotional mobility and suspicion characterize the mental phenomena of cardiac disease.

D'Astros has observed that the patients with aortic disease display very frequently an extreme impressionability, a variable, capricious, and fantastic character, and exaggerated susceptibility. The patients with mitral lesions are somewhat self-contained, and reticent. They are depressed, taciturn, and manifest a *tædium vitæ*, and are at times plunged into extreme depression. Mildner has reported cases in which cardiac insufference were accompanied by emotional exaltation, followed by suspicion and furor.

Sibson says "that the chief psychical symptoms presented are depression; sometimes the patient was taciturn; sometimes having hallucinations, and sometimes displayed lypemaniac frenzy."

Morel has observed in patients attacked by paroxysmal dyspnœa resulting from cardiac disease, the periodical return of strange ideas, hypochondriacal sensations, and hallucinations of a depressing nature. Krapelin states that disease of the heart exerts a peculiar influence on the types of insanity which occur during acute rheumatism, causing them to be of an emotional, suspicional type.

Spitzka holds that the view that cardiac hypertrophy of the left side with aortic valvular lesion is more apt to be associated with maniacal conditions, and cardiac hypertrophy of the right ventricle with mitral valvular lesion with melancholic states, is supported by too few observations to merit acceptance; the blood pressure in recorded cases was not registered, hence these are destitute of value. The heart has important and direct relations to the brain, and it is very likely that just as disturbances of the vagus inervations are responsible for the *raptus melancholicus*—in other words, just as a disordered state of the brain reacts on itself through the medium of the functional cardiac influence it produces—so a valvular lesion may directly influence the emotional states without pre-existing brain trouble. It is a fact that patients suffering from cardiac lesions are more likely to develop anxious and suspicious delusions than those of an opposite nature. Forbes Winslow expresses similar opinions. My own experience agrees with D'Astros and Griesinger in regard to the influence of cardiac disease on insanity. Spitzka very correctly says that the mental phenomena produced by it furnish a rich pabulum for delusion.

That insanity produces cardiac disease is demonstrated by the experience of Salemi—Pace and Milner Fothergill to point out that in states of emotional exaltation cardiac murmurs, especially of the aortic valves, are frequent without valvular lesion.

Griesinger points out that in conditions of exaltation murmurs, particularly of the aortic valves, are very frequent without coincident valvular lesion. He states that observations in the Vienna asylum show that during maniacal conditions the heart sounds are indistinct during great excitement, and become clear when calm sets in.

My own experience is not only in accord with this, but I have also noticed that in the cataleptoid state of katatonia temporary murmurs result. These facts have a direct bearing on the prognosis of the disease. Mental and physical quiet is urged on almost every sufferer from cardiac disease. For a long time such advice is observed. The suspicional state raising from the disease and the long continued relief from untoward symptoms resulting from this rest predisposes the patient to take a different view of the state of things from his physicians. When emotional exaltation results from disease, there are but few checks on the patient to restrain his indulgence in exuberant horse-play. These features are well illustrated by the case of Matthew Arnold. Matthew Arnold came from a well-balanced English family, one of those old English families who have learned to restrain emotion. His father, the head of the famous school at Rugby, which Tom Hughes has immortalized, was also a sufferer from cardiac disease, but old Dr. Arnold was very favorably situated; he was in a position to lead a regular, uneventful life. His course of study and course of life was laid out for him, and he was held in high esteem by those by whom he was surrounded. For that reason he died well advanced in years and universally regarded as a gentleman of peculiar sweetness of temper, amiability, and exactness in all particulars. Many of these traits were noticeable in his son, Matthew Arnold, who has just died. He called himself peculiarly the "Apostle of Sweetness and Light."

He preached to a certain extent a philosophy somewhat modified from the theory of the quietest philosophy, which was practically a survival of Buddhism. There is little doubt but that admiration of this restraining philosophy, which Arnold adopted to a certain extent (as did Cowper, another man of letters who was demonstrably insane) had good results. Matthew Arnold's disease improved under the influence of this philosophy which tended to restrain all emotional display. His training also restrained all emotional peculiarities as "bad form." For a long time Matthew Arnold was in many respects a model man. About twenty-five years ago he contracted heart disease in consequence of a rheumatic attack which affected the aortic valve, and I believe there finally developed a mitral lesion, too, but of that I am not certain. The aortic valve was affected, and he had aortic stenosis. Through the advice of Sir Andrew Clark he led for a long time a comparatively quiet life. He devoted himself to subjects in literature that would tend rather to restrain the emotional element than to develop it. On the other hand, of late years he had been less restrained. In point of fact it was one of these emotional displays that alarmed Sir Andrew Clark and made him lay the injunction on Matthew Arnold to lead a restful life. At times his disposition was anything but sweet; some of his poems, one of which has been republished recently, in dealing with the medical and clerical professions, manifests a cynicism which would not discredit Swift.

This cynical mood was purely spasmodic, and it was often replaced by one in which his title of Apostle of Sweetness was well deserved. It is noticeable that the same period of buoyancy was succeeded afterwards by one in which horse-play was a distinguishing characteristic of the apostle. In the period preceding his death these alternations were singularly frequent and well marked. He had been exceedingly bland and buoyant. Soon after he decided to show his young friend his manly agility, attempted to jump over a gate and fell dead.

The bearing of these facts on prognosis is simple and direct. Mitchell Bruce has pointed out that the prognosis of cardiac disease in what he calls "nervous cases" (which include cases where emotional mobility is well marked), is unfavorable.

The cardiac disease but too often produces these emotional states, and they aggravate the cardiac disease. Given, therefore, a case of cardiac disease in which emotional displays occur frequently these last indicate moral treatment; and furthermore indicate a very unfavorable prognosis, more especially since, as already stated, too often suspiciousness occurs resulting in distrust of the physician and of remedial measures. These frequent perturbations cause a nutritive change in the heart, and as Bruce, A. Mickle, and Salemi-Pace have shown, are very important factors in the production of cardiac weakness.

The point on which I desire to lay stress is that this great buoyancy which occurs in the course of these troubles is as much a part of the disease as the murmurs, and furthermore, instead of feeling encouraged by the patient's buoyancy and good spirits an effort should be made to restrain them as much as possible. This emotional factor in most ordinary disease is a good deal ignored; and this is particularly true of cardiac disease, though by the older authors, Griesinger and Watson, attention is called to these facts. A good deal could be done by moral measures to restrain these emotional displays, and by remedial measures, and control of the heart's action by the proper use of certain cardiac tonics. A case of cardiac disease in which these emotional phenomena occur with great frequency, is very likely to have a bad prognosis, since the emotional condition is produced by the cardiac disease; and, on the other hand, these recurrences of emotional excitement are more and more likely to be followed by cardiac failure and degeneration. For this reason I desire to call attention to this somewhat neglected phase of disease.

Dr. ROBERT H. BABCOCK: I regret exceedingly not having heard the first part of the paper, and so rise with some hesitation, but I would like to ask the author whether he attributes the mental symptoms to the cerebral hyperæmia produced by the heart disease; or whether, perchance, to faulty digestion and faulty elimination, since the liver and kidneys are interfered with in their functions from a passive hyperæmia set up by these heart cases. In my experience with these cases I have found the mental state of the patient to depend very largely upon the state of the liver—digestion, so to speak—and the mental state is relieved by measures which will relieve the engorgement. I find the alternation of mood, melancholy, hilarity, etc., relieved by regulation of the diet, and I have had this fact especially impressed upon my mind within the past two months by a case of advanced heart lesion, in which, owing to the great enlargement of the liver I decided to cut off all nitrogenous diet from the patient—that is, in the way of meat and eggs, and limit her to a vegetable and farinaceous diet with milk, and as a result the improvement has been exceedingly marked, and is noticeable in the mental state as well as in the physical. I would like to hear the opinion of the author on this subject. It seems to me that if we paid more attention to the proper nourishment of the patient, with reference to the ability of the digestive organs to carry out digestion to its fullest extent, instead of stopping short at the production of uric acid, we could do much more to regulate those alternations of mood.

Dr. N. P. PEARSON: I would ask if the urine had been examined in this patient? I think it is necessary wherever we wish to make a complete diagnosis. In heart disease there is likely to be passive hyperæmia of the liver and kidneys, and our pathology teaches that biliary acids and renal matter are liable to derange the brain, and produce all the symptoms spoken of. I always insist upon a careful examination of the urine in such cases in order to distinguish between hyperæmia of the brain and a disordered state of the liver and kidneys.

Dr. KIERNAN, in closing said: The conditions referred to by Drs. Babcock and Pearson, while deserving of attention, hardly come under the category of the mental phenomena to which I desired to call attention. They are more permanent and less fluxionary.

The use of urinalysis, while of value in the permanent type, is rather too gross a test to be applied in the rapid fluxionary types.

BAYARD HOLMES, M. D., read a paper entitled "Secondary Mixed Infection in Typhoid Fever," which appeared in the August number of this journal.

Dr. CLARK GAPIN: I rise to acknowledge my sense of obligation to Dr. Holmes. He has in the most skillful way written up a subject that is to me of great interest. I would like to ask that in his summing up he will state what, in his study of typhoid fever, is the best means of preventing the attacks of these little monsters of which he has so interestingly written, especially as they come from water. I believe that it is stated that the greatest source of danger is water; probably greater than all other sources combined; especially the water of large cities. I hope the author will suggest some means of treating water that will be certainly destructive of all germs.

Dr. HENRY GRADLE: It seems to me that the Society owes Dr. Holmes a little more recognition than to accept his paper in silence. As far as I am aware, this is the first attempt to trace clinically the history of a disease as a series of successive infections which has hitherto been considered as an etiological unit. The light which the address has thrown upon the subject seems to me to illuminate the clinical points more elaborately than microscopical research has as yet done. He has based his paper on analysis, and in many cases on inferences of such a positive nature that I believe, from a clinical point of view, a great deal of information can be admitted as correct, although not fully substantiated as yet by the necessary microscopical research. This question of mixed infection will probably take a greater place in our views of pathology than has hitherto been accredited to it. I believe it is not difficult to point out a large number of instances where there can be no doubt about the clinical unity consisting of several separate factors, although unlike typhoid fever, the actual proof is wanting. Take, for instance, small-pox, or simply cow-pox. Up to the present time I believe it is generally admitted that the etiological factor productive of these two diseases has not yet been determined; we are not yet in a condition to say which parasite causes small-pox and cow-pox, but we know both of these diseases have a certain clinical source, the latter part of which is entirely due to micrococci, the familiar staphylococci of suppuration. These have been isolated, and with them we can reproduce certain features common to small-pox and cow-pox, but we cannot reproduce the original diseases. They are the scavengers, so to speak, that follow the original parasite, but can be separated from them by culture. Whenever we vaccinate an individual we get not only the cow-pox pustule, but the ultimate pustule due to micrococci of suppuration. Similarly, diphtheria is due not to one parasite alone; the original parasite, the identity of which is not absolutely established, does not enter into the deeper tissues; it is limited in its ravages to the mucous membrane, which it invades, but the secondary stage is due to septicæmia from micrococci infection. It is the same in a great many other internal diseases, the parasites of which have not been recognized with certainty, while we are acquainted with the causes of septicæmia accompanying them, and representing a secondary infection.

Professor Frank Billings: I want to add my mite of thanks to Dr. Holmes for this most excellent paper. Some time ago, when I learned from Dr. Holmes that he was going to write on this subject, I called to mind several cases that illustrate his paper.

While an interne in Cook County Hospital, during August, September, and October, 1881, there were over one hundred patients daily in the hospital suffering from typhoid fever, and quite a number of these cases illustrated well the subject of Dr. Holmes' paper. Two or three cases were so emphasized to my mind that I remember them quite well. One, a young Swede about twenty-four years old, suffered from a severe form of typhoid fever three or four weeks. During convalescence he had left iliac phlebitis with secondary fever, and subsequently he had at least twenty subcutaneous abscesses all over his body—on the back, abdomen, thighs, and also an abscess of one middle ear. He had, in other words, a pyæmia, and it seemed to result from that thrombosis of the left iliac vein, for suppuration resulted there primarily. He was removed from that ward into the infectious portion of the hospital, so that I did not see him again. But he finally recovered under proper care.

I remember another case, a young American twenty-one years of age. He was sent to the hospital suffering from a very severe form of typhoid fever, and in his convalescence he suddenly developed subcutaneous hæmorrhages all over the abdomen and on the inside of the thighs. He must have had capillary emboli, and of course they must have come from some infection, whether you call it bacteria, or what not. Small abscesses resulted wherever these little spots appeared; the hæmorrhage became diffused in places, but the suppuration was never excessive.

In another case secondary unilateral empyæma occurred. I could give other cases, but these are sufficient to serve as examples of what Dr. Holmes has so ably shown us to be a secondary infection in typhoid fever.

Dr. HOLMES, in closing the discussion, said: I wish to thank the Society for the reception given this paper, and to say that I have attempted no discussion of the etiology or treatment of this well-known disease. I must admit that the remarks of Dr. Gradle contained the whole truth. The deductions in this article are rather more extensive than our knowledge of the subject will at present permit, but I have every confidence in the verification of most, if not all, of my statements, by subsequent investigation.

